# Measuring organizational readiness for knowledge translation in chronic care

**DOI:** 10.1186/1748-5908-6-72

**Published:** 2011-07-13

**Authors:** Marie-Pierre Gagnon, Jenni Labarthe, France Légaré, Mathieu Ouimet, Carole A Estabrooks, Geneviève Roch, El Kebir Ghandour, Jeremy Grimshaw

**Affiliations:** 1 Research Center of the Centre Hospitalier Universitaire de Québec, Québec, Canada; 2 Faculty of Nursing, Université Laval, Québec, Canada; 3 Department of Family Medicine, Université Laval, Québec, Canada; 4 Department of Political Science, Université Laval, Québec, Canada; 5 Faculty of Nursing, University of Alberta, Edmonton, Alberta, Canada; 6 Ottawa Hospital Research Institute, Ottawa, Canada; 7 Faculty of Medicine, University of Ottawa, Ottawa, Ontario, Canada

## Abstract

**Background:**

Knowledge translation (KT) is an imperative in order to implement research-based and contextualized practices that can answer the numerous challenges of complex health problems. The Chronic Care Model (CCM) provides a conceptual framework to guide the implementation process in chronic care. Yet, organizations aiming to improve chronic care require an adequate level of organizational readiness (OR) for KT. Available instruments on organizational readiness for change (ORC) have shown limited validity, and are not tailored or adapted to specific phases of the knowledge-to-action (KTA) process. We aim to develop an evidence-based, comprehensive, and valid instrument to measure OR for KT in healthcare. The OR for KT instrument will be based on core concepts retrieved from existing literature and validated by a Delphi study. We will specifically test the instrument in chronic care that is of an increasing importance for the health system.

**Methods:**

Phase one: We will conduct a systematic review of the theories and instruments assessing ORC in healthcare. The retained theoretical information will be synthesized in a conceptual map. A bibliography and database of ORC instruments will be prepared after appraisal of their psychometric properties according to the standards for educational and psychological testing. An online Delphi study will be carried out among decision makers and knowledge users across Canada to assess the importance of these concepts and measures at different steps in the KTA process in chronic care.

Phase two: A final OR for KT instrument will be developed and validated both in French and in English and tested in chronic disease management to measure OR for KT regarding the adoption of comprehensive, patient-centered, and system-based CCMs.

**Discussion:**

This study provides a comprehensive synthesis of current knowledge on explanatory models and instruments assessing OR for KT. Moreover, this project aims to create more consensus on the theoretical underpinnings and the instrumentation of OR for KT in chronic care. The final product--a comprehensive and valid OR for KT instrument--will provide the chronic care settings with an instrument to assess their readiness to implement evidence-based chronic care.

## Background

Organizational changes are becoming increasingly important in the present healthcare environment, with an emphasis on long-term management of chronic conditions [1,2]. According to current estimates, one-third of the Canadian population is affected by one of the six most common chronic conditions, namely, heart disease, chronic obstructive pulmonary disease (COPD), diabetes, mood disorders, cancer, and arthritis [3]. However, the implementation of evidence-based recommendations on optimal chronic care into various clinical settings has been incomplete, highlighting the difficulty to translate knowledge to the concrete care context [4]. Therefore, important 'care gaps,' *i.e*., a difference between best care and usual care, have been reported in the case of all chronic diseases covering access, diagnosis, prescription, and treatment adherence [1]. For example, in the case of diabetes, even if several efficient strategies to prevent or delay diabetes complications exist, these strategies are suboptimally implemented in practice [5]. Fewer than one-half of the patients receive the recommended lab tests and procedures to prevent serious complications [6]. Also, among Canadians suffering from heart disease, only 50% receive proven therapies on a regular basis [1]. While organizational context has been shown to influence research utilization in practice [7,8], healthcare organizational members and structures still need to have a sufficient readiness for implementing research-based knowledge.

As argued by the World Health Organization (WHO), 'to address the rising rates of chronic conditions, an evolution in health care systems is imperative, and they have to advance beyond the acute care model' [9]. Consequently, changes to chronic care delivery that aim at organizational, systemic factors in the healthcare system are increasingly promoted by health researchers [1,2,5,10]. The Chronic Care Model (CCM), developed by Edward H. Wagner *et al. *at the MacColl Institute for Healthcare Innovation, is a well-known conceptual model of the primary elements crucial for managing chronic conditions. It is shown that focusing on chronic care should imply a systemic approach based on planned, proactive care organized around the interactions between the patient and an integrated practice team [11]. Also, it should rely on best evidence that is applicable in different facets of the care system identified with the CCM.

Organizational characteristics have been associated with healthcare professionals' motivation to improve quality of chronic care [12,13]. On the other hand, various aspects related to the organizational context and climate (e.g., collaborative decision - making, strong leadership, committed financial and corporate support, strengthened communication and infrastructure) have proven to facilitate the implementation of CCM elements [3]. 'Implementation needs the engagement of management and enough resources at the grassroots level to take care of all tasks (i.e., acute and chronic care)' [3]. Thus, organizations need to be both equipped and motivated to integrate new research-based knowledge on optimal chronic care in the practice. In other words, they have to have a sufficient level of readiness to a research-informed change.

The quality improvement process should be built on planned and scientifically informed knowledge translation (KT) interventions ensuring that the knowledge users are aware of, have access to, and can use the research evidence to inform their practices related to managing chronic conditions. 'These initiatives must include all aspects of care, including access to and implementation of valid evidence and organizational and systems issues' [14].

In this project, we focus on identifying, appraising, and testing measures of organizational determinants in KT in chronic care services. We are particularly aiming at assessment tools based on theorizing about organizational readiness (OR) that would be used to assess an organization prior to implementing evidence-based and scientifically-informed knowledge related to the core elements of the CCM.

### The chronic care model

According to current available knowledge, the CCM developed by Wagner *et al. *provides a synthesis of evidence-based system changes needed for improving chronic care [15]. The CCM, originally created within the US national program Improving Chronic Illness Care (ICIC) in 1998, has informed chronic care redesigns in numerous health organizations. It is an internationally applied model that has also served as a basis for the development of complementary CCMs, such as the WHO's Innovative Care for Chronic Conditions (ICCC) framework [9] aimed at global health policies, and the more health promotion-oriented Expanded Chronic Care Model (ECCM) [16]. According to recent reviews, the application of CCM has shown evidence of quality improvement in the processes and outcomes in managing various chronic conditions such as diabetes, asthma, heart failure, and depression [15,17,18].

The creation of the CCM was based on evidence from scientific literature describing practice innovations and interventions associated with improved healthcare and outcomes [19]. It is developed based on an extensive literature review on best practices, expert opinion, and comparison between quality improvement interventions in chronic illness management [20]. The model was created with the objective of bridging the gap between best care and usual care in the context of rising burden of chronic conditions. The CCM is intended as an 'evidence-based guideline' offering synthesized knowledge of the best available evidence to guide quality improvement initiatives and disease management activities related to chronic care [2].

**Figure 1:**
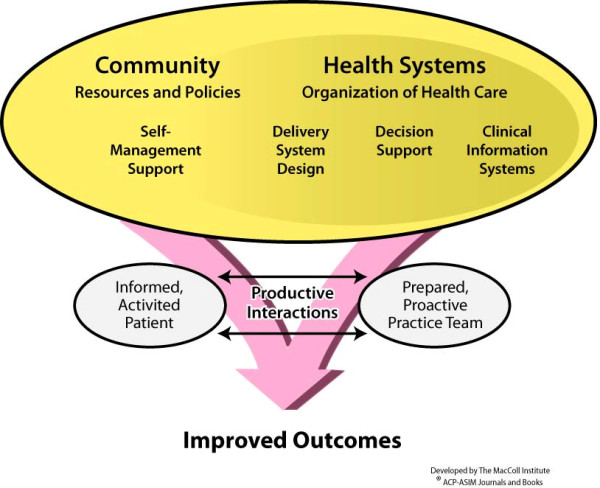
**The Chronic Care Model **(**The MacColl Institution for Healthcare Innovation)**.

According to the CCM, improved care processes and outcomes can be achieved by six interrelated system changes that support 'the development of informed activated patients and prepared proactive healthcare teams whose interactions become more productive and satisfying around chronic illnesses' [17] (Figure 1). These components include healthcare organizations linking with community resources and policies with the organizations' main focus on four system components, namely: delivery system design, decision support, support for self-management, and clinical information system [15,17]. As the evidence of the CCM shows, improved chronic care therefore requires multiple systemic changes and, consequently, a sufficient level of OR for KT that is needed for implementing changes in different facets of the care system.

### Assessing organizational readiness for change: conceptual and empirical challenges

Health services researchers have only recently begun to theorize about developing measures of organizational readiness for change (ORC) and to empirically assess it, although this concept has been recognized for some time [21]. In their extensive review, Weiner *et al. *[21] examined how ORC has been defined and measured in health services and in other fields. Through the analysis of 106 peer-reviewed articles and the assessment of 43 instruments, they identified some conceptual and methodological issues that need to be addressed for measuring ORC.

First, Weiner *et al. *noticed little consistency with regard to conceptual terminology and the meaning of OR. Seventy-seven percent of the articles reviewed by Weiner *et al. *used alternatives to the term 'readiness for change' (*e.g*., preparedness or willingness), and only one-half of the articles provided some kind of definition of ORC [21]. Also, two general approaches, psychological and structural, were found in describing readiness for change, and hence, the level of analysis varied from individual or organizational to the combination of both.

Second, the review by Weiner *et al. *also brought up the limited evidence of reliability and validity of most currently available instruments. Only seven instruments from the total of 43 reviewed measurement tools had undergone a systematic assessment of validity and reliability [21]. The lack of validity and reliability of the existing ORC measures is also confirmed by Holt *et al. *[22]. By reviewing the literature on ORC measurement instruments, they systematically classified and described 32 different instruments assessing OR. Only two of the 32 instruments--Burke *et al.*'s Lay of the Land Survey [23] and McConnaughy *et al.*'s URICA [24]--showed evidence of content, construct, and predictive validity [22]. The study by Holt *et al. *also showed more global discrepancies in the operationalization of ORC. Even if several factors were included in ORC measures, the literature review revealed a lack of comprehensive assessment of readiness for change [22].

Weiner *et al. *conclude that the content of an OR construct must include two approaches identified in the literature, the first describing ORC in psychological terms (organizational members' attitudes, beliefs, and intention), and the other describing ORC in structural terms (emphasizing organizational capabilities and resources) [21]. In his recent publication theorizing ORC, Weiner combines the psychological and structural dimensions by defining OR as 'a shared psychological state in which organizational members feel committed to implementing an organizational change and confident in their collective abilities to do so' [25]. Weiner also states that OR is a multi-level and heterogeneous construct in that 'the construct's meaning, measurement, and relationships with other variables differ across levels of analysis' [25].

Supporting the observations made by Holt *et al. *[22] in their review of instruments measuring OR among public and private sector organizations, Weiner *et al. *[21] conclude that researchers need to give greater attention to measurement development, testing, and refining. A comprehensive assessment of ORC should embrace two determining factors (psychological and structural) operating in two different levels (individual and organizational) [26]. In line with the conclusions of Weiner and Holt, Walker *et al. *conclude that 'a complete model of [organizational] change should address not only macro-level forces such as content, process, and contextual factors, but also micro-level factors such as individual differences.' [27]

Following the discussion of Weiner *et al. *on the theoretical composition of ORC, we consider ORC as a multidimensional construct covering both the psychological (*i.e*., motivational) aspects as well as the structural factors related to human and technical resources. It is hypothesized that chronic care organizations' readiness affects the process of translating knowledge related to one or several aspects of optimal care described in the CCM. Despite the identified conceptual and empirical challenges, ORC remains an appropriate evidence-based concept to be operationalized for the assessment of organizational capacities to engage in a KT change regarding chronic care.

### Knowledge translation to improve chronic care

KT, as defined by the Canadian Institutes of Health Research (CIHR), is a dynamic and iterative process that includes the synthesis, dissemination, exchange, and ethically sound application of knowledge to improve health, provide more effective health services and products, and strengthen the healthcare system [28]. KT in healthcare services is influenced by factors at different levels of the healthcare system. These levels include individual healthcare professionals, healthcare team, healthcare organization, and broader healthcare system [8,29-38]. However, up to now, KT strategies have been mainly targeted at the level of healthcare workers [39]. As these strategies appear to be insufficient for changing healthcare professionals' performance [40] and influencing patients' outcomes, other elements, such as contextual or organizational factors, must be taken into consideration [38,41-45].

In order to explore OR for KT in healthcare services, we need to identify and apply valid measures of key determinants of KT. Considerable progress has been made in exploring the impact of individual healthcare professional factors on KT by applying social cognition models from health psychology [29,30,33,36,37]. Also, the influence of organizational factors on KT is largely recognized. Multiple type of organizational factors influencing KT have been studied, including such aspects as organizational complexity, centralization, size, presence of a research champion, traditionalism, organizational slack, time constraints, access to and amount of resources, professional autonomy, and organizational support [46]. Furthermore, considerable work has been done in assessing the influence of healthcare organizational context in evidence-based practices [38,47,48].

**Figure 2:**
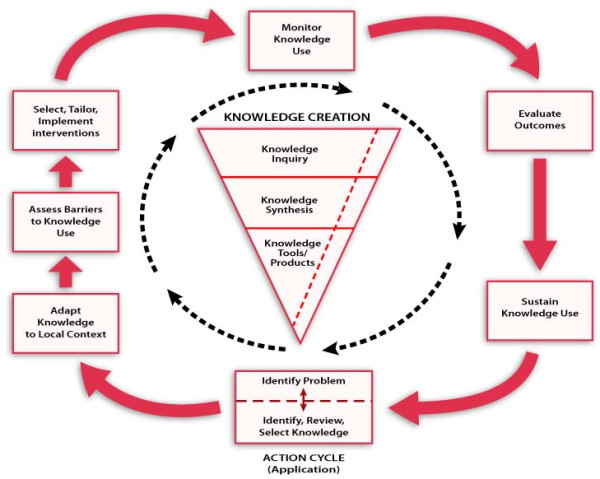
**Knowledge to action process (Graham, *et al. *2006)**.

This study will shed light to the role of organizational factors in the knowledge-to-action (KTA) process where the implemented knowledge is research-based. The KTA framework elaborated by Graham *et al.*, conceptualizes KT as an iterative, dynamic, and complex process comprising knowledge creation and knowledge application [14] (Figure 2). Knowledge creation comprises three phases: knowledge inquiry, knowledge synthesis, and creation of knowledge tools. Knowledge application (action cycle), which is the main interest of this study, includes: identifying the problem; adapting knowledge to local context; assessing barriers and facilitators to knowledge use; selecting and implementing interventions; monitoring knowledge use; evaluating outcomes; and sustaining knowledge use. However, the action cycle is influenced by knowledge creation, and several action phases can take place simultaneously.

The application of the KTA framework in this project is relevant for two reasons. First, as one of the challenges of the CCM is its vagueness on the specific care process changes to adopt and on the ways to achieve them [49], we need to choose an appropriate implementation approach, such as provided by KTA, and apply it for specific KT interventions. Second, KTA highlights the role of the end users of the knowledge in the translation process, hence making sure that the knowledge is both relevant and applicable for the specific context [14].

### Objectives

Our aim is to assess the influence of OR for KT in chronic care. We therefore need to: identify the key facets of OR relevant for the KTA process; validate these key facets in different chronic care contexts; and develop a valid and comprehensive instrument to measure their influence in KT.

Given the lack of consensus on the theoretical foundations and the instrumental properties of ORC, our first objective is to systematically review the literature on conceptual frameworks, theoretical models, and instrumentation of ORC to identify the core concepts to be operationalized for measurement with a KT approach.

In order to facilitate the identification of an appropriate ORC measuring instrument by different stakeholders, our second objective is to produce a database of instruments for measuring ORC that could be applied to KT in the healthcare sector. This database will incorporate key information about the properties of the instruments and the relevance for assessing OR for different steps of the KTA process and for different types of organizations (*e.g*., acute care, long term facilities, and community health). It would also provide summaries for use by decision makers and policy makers.

Third, the systematic review findings will be validated by means of a Delphi study in order to prioritize the concepts and measures that will be retained for further instrument development.

Our final objective is to develop, validate, and apply a comprehensive integrated instrument to gauge OR for KT in a sample of chronic care organizations. The instrument will first be validated in chronic care services in the province of Quebec, Canada, and then tested in three Canadian provinces in chronic disease management to measure OR regarding the adoption of evidence related to comprehensive, patient-centered, and system-based chronic care. The testing of this consensual OR for KT measurement tool will help decision makers to get a picture of the motivation and capacity of their organization to implement specific innovations in chronic care based on the best scientific evidence available.

## Methods

### Phase one: Systematic review of ORC literature and Delphi study

We will conduct a systematic review of the theories and instruments assessing OR to adopt new knowledge and implement a change at the organizational level. We will identify and appraise the psychometric properties of different measurement instruments to populate the identified domains building upon recent syntheses of organizational determinants of innovation and KT [50,51]. This systematic review will focus on specific domains, concepts, and items of OR related to KT identified by decision makers and experts in organizational change theories.

### Study identification

We will perform broad searches across the health sector to identify theoretical and empirical studies on ORC that either describe a theory, model, or framework of OR related to KT or report the use or testing of an ORC measurement tool. Standardized literature searches will be conducted on all relevant databases (MEDLINE, Pubmed, Ovid, Cochrane Central Register for Controlled Trials, Campbell Collaboration Register for Controlled Trials, Current Content, Science Citation Index, Social Sciences Citation Index, LISA, CINAHL, PsychINFO, EMBASE, ProQuest). Any relevant references from studies found through the above routes will be followed up and obtained for assessment. All team members will be asked to search for relevant articles published in their specific field. We will also search for appropriate grey literature through internet search engines and on governments' websites.

### Inclusion and exclusion criteria

Quantitative, qualitative, and mixed-methods designs will be considered since the focus of this review is to identify relevant components of OR to be operationalized for a KT approach to change. However, instruments with closed-ended questions and response formats allowing psychometric assessment will be of specific interest for the further instrument development. Studies published in English, French, Spanish, Finnish, or Swedish will be included. Only cases referring to the healthcare domain and applying the concept of ORC or equivalent terms (*e.g*., preparedness, commitment, or willingness to change) will be reviewed. The retrieved documents have to relate to a theory, a theoretical component, a model or a framework. Purely theoretical papers on ORC and applicable in the healthcare domain will also be considered, but editorials, commentaries, and checklists will not be eligible for inclusion.

### Study selection

All titles and abstracts will be screened independently by one of the investigators and a research professional to assess which studies fit the inclusion criteria. Any discrepancies between the two reviewers on study inclusion will be resolved by discussion with other team members. Full text copies of all potentially relevant papers will be retrieved. Then, each study will be independently abstracted and appraised by two reviewers randomly chosen among the team members.

### Data extraction

A critical appraisal of all included studies will be conducted to compare the nature and the scope of conceptual models, frameworks, or theories on organizational factors influencing KT (*e.g*., origins, similarities, differences, inclusiveness) as well as their strengths, limitations, and the extent to which they have been tested in the field of health services organization.

### Appraisal of study quality

The quality of all eligible studies will be assessed by the two independent reviewers using quality criteria specific to quantitative, qualitative, and mixed-methods designs [52]. Studies that do not meet a minimal quality threshold on their respective quality scales will be excluded. Any discrepancies in quality ratings will be resolved by discussion and involvement of an arbitrator among other team members when necessary.

### Methods for synthesizing findings

The findings of the systematic review will be synthesized and represented graphically by the means of a conceptual map created with the CmapTools software kit developed by the Institute for Human and Machine Cognition (IHMC) [53]. Conceptual maps have been proved efficient in capturing and sharing expert knowledge [53]. They enable organizing and connecting knowledge in a hierarchical and interrelated manner. They also facilitate new knowledge creation, which can be characterized as 'a relatively high level of meaningful learning accomplished by individuals who have a well organized knowledge structure in a particular area of knowledge, and also a strong commitment to persist in finding new meanings' [54].

The conceptual map will synthesize knowledge on the different components of ORC. We will seek to reveal five components by the mapping. We will capture the various dimensions of ORC described by the identified theoretical models, as well as the strengths and weaknesses of these models. We will also identify the outcomes of OR. We will represent the knowledge on the level of analysis used to measure OR. Finally, the map will synthesize the information on the operationalization of the ORC dimensions as instrument items. This mapping will serve as a basis for the development of the OR instrument with enhanced validity. This conceptual map will be inspired by the work of Weiner et al. who suggest a classification of the core elements of ORC [25].

We will then assess ORC instruments with an existing checklist for assessing psychometric properties using the Standards for Educational and Psychological Testing [55]. Finally, we will prepare a bibliography and a database of these instruments for the use of researchers and decision makers in different healthcare organizations.

We will propose a classification of OR instruments based on the various steps of the KTA cycle proposed by Graham *et al. *[56]. As such, organizational factors potentially influencing readiness for KT will be presented according to their possible impact on one or several of the seven steps identified in the KTA cycle: identify problem, adapt knowledge to context; assess barriers and facilitators to knowledge use; select and implement interventions; monitor knowledge use; evaluate outcomes; and sustain knowledge use. This will be a unique contribution of this review.

### Delphi study on organizational factors influencing KT

In preparation for the Delphi study, we will convene a panel of academic experts on theories and measures about organizational change and KT to identify concepts of OR that may impact KT. These concepts and measures will be identified from the systematic literature review on ORC. Then, an online Delphi study will be conducted among decision makers and knowledge users across Canada to assess importance of these concepts in their contexts. The aim of the Delphi study is to obtain opinions from groups representing a variety of expertises and contexts in order to adapt our final OR instrument to the Canadian primary healthcare context. The Delphi study is considered to be a strong methodology for a rigorous consensus of experts on a specific theme. Usually, between 10 and 18 experts are needed in the process [57]. Recruitment of experts will be done through the contacts network method [58], with the help of team members and their extensive network of collaborators.

Delphi participants will be asked to rate the relevance, the applicability and the importance of each proposed items on a seven-point Likert scale (*e.g*., 1 = not relevant to 7 = extremely relevant). They will also be able to add free text comments. Results from the first round will be compiled and a mean score of the parameter (*e.g*., relevance) will be calculated. Then, participants will be invited to take part in a second round of rating. Participants will again be asked to rate the degree of relevance of each of the identified factors. This survey will also show the first round ratings by providing the mean score for each item. Reminders will be sent to participants after in each round. Then, consensus will be sought for each proposed measure of organizational factors (a 70% agreement rate is considered consensual [57]). Only measures for which a consensus is reached will be kept, after the second or third round, if necessary.

Based on the systematic review and the Delphi study, a final mapping of the constructs of organisational readiness for KT in chronic care and available measurement instruments will conclude Phase one.

### Phase two: ORC instrument refinement, validation, and application

Based on the results Phase one, we will develop a comprehensive instrument to measure OR for KT. A preliminary version of the instrument will be prepared in both French and English. In order to meet the needs of the contemporary healthcare organizational environment, the questionnaire will be developed with a specific concern to gauge OR for adopting complex, system-based interventions to be applied in multidisciplinary healthcare contexts. Preparation work will be done in advance of the validity testing, including determining access to appropriate health center data and relevant accreditation data, and developing sampling frames. Following the recommendations derived from the reviewed evidence, this measurement instrument will embrace both psychological and structural determinants on the organizational level [26,27].

### Testing instrument validity: Implementation of evidence-based chronic care

The comprehensive measurement tool will first be assessed using feasibility testing in a purposive sample of healthcare organizations from three Canadian provinces (Alberta, Ontario, Quebec) during the third and fourth years of the project. Prior to testing the OR instrument, we will identify a relevant 'KT case' on quality improvement in chronic care for each province that will allow us to test the developed questionnaire in both French and English versions in different care contexts. The developed instrument will be explored in chronic care services to measure healthcare organizations readiness to implement research evidence related to adopting integrated, systemic, and patient-centered CCMs.

The field testing of the developed questionnaire will follow the standards for educational and psychological measurement that propose a set of criteria regarding test construction, evaluation, and documentation [55,59]. Because the questionnaire will be self-administered, we will also obtain data from healthcare organization accreditations (*e.g*., Accreditation Canada) and recent reports from provinces that are involved with their use, including Ontario and Quebec. These data will be used since they are easily available and offer comparison standards (*e.g*., Qmentum) for various organizational aspects that have been measured in an objective manner. The fifth year of the project will allow us to complete the data analyses and for knowledge transfer activities.

### Ethical considerations

Exemption from ethics approval for the first phase of the project has been received from the Research Ethics Board of the Centre Hospitalier Universitaire de Québec (November 10, 2010; ethics number S10-12-113). Ethics approval will be requested from the Research Ethics Board of the Centre Hospitalier Universitaire de Québec for the second phase of the project that includes conducting individual interviews and focus groups, as well as from other healthcare organizations that will participate in the field study.

Participants in the Delphi study and stakeholders recruited for the individual interviews and focus groups will be sent a specific consent form that presents the research objectives and information about research implications. They will be informed that participation in the research is entirely voluntary. With regard to the Delphi study, the participants will be informed that their consent is implicitly confirmed when creating their electronic account.

### Deliverables

The deliverables for this project include: a systematic critical appraisal of theories/models/frameworks on factors influencing OR for KT and related measurement tools synthesized in a concept map; a set of core measures for assessing OR for KT that will be available in a database and a searchable website; and a validated OR for KT change tool adapted for Canadian healthcare settings and for services planning to implement research-informed changes related to chronic care improvement. Following each phase of the research, scientific manuscripts will be prepared and submitted to open access scientific journals. Also, plain language summaries will be disseminated to various stakeholders groups, such as national and provincial health ministries, healthcare professional associations, and healthcare organizations networks. At the end of the project, a 1-3-25 format report will be prepared and sent to key stakeholder groups. KT Canada's website and conferences will provide avenues to disseminate the project's results to academics, decision makers, policy makers, and the general public.

## Discussion

This study will provide an assessment tool to measure healthcare organizations' readiness for KT, described as a KTA process. The instrument development will be based on a comprehensive synthesis of current knowledge on organizational characteristics affecting readiness for KT change in healthcare services. The literature findings will be further validated by the Delphi study which will enable us to contextualize the findings in Canada for further instrument development and refinement. With the elaboration of OR instruments, database, and website, this research will also provide useful tools for stakeholders and decision makers in assessing their organizations' readiness for successful knowledge implementation. The collaboration with key stakeholders and decision makers in developing the comprehensive readiness instrument will promote the application of the research findings in various health services contexts. By validating the OR for KT instrument in a sample of chronic care organizations, the project aims to support the development of enhanced systematic interventions to meet the needs of the contemporary healthcare setting.

## Competing interests

The authors declare that they have no competing interests.

## Authors' contributions

All authors collectively drafted the research protocol and approved the final manuscript. MPG is the principal investigator and should be contacted for further information on this research project.
